# Double-chambered left ventricle: a rare incidental finding on echocardiography

**DOI:** 10.1093/ehjci/jeaf103

**Published:** 2025-04-02

**Authors:** Tuomas Laalo, Riikka Uotila, Valtteri Uusitalo

**Affiliations:** Clinical Physiology and Nuclear Medicine, Helsinki University Hospital and University of Helsinki, Stenbäckinkatu 11, 00290 Helsinki, Finland; Clinical Physiology and Nuclear Medicine, Helsinki University Hospital and University of Helsinki, Stenbäckinkatu 11, 00290 Helsinki, Finland; Clinical Physiology and Nuclear Medicine, Helsinki University Hospital and University of Helsinki, Stenbäckinkatu 11, 00290 Helsinki, Finland

A 70-year-old Caucasian woman was referred to the transoesophageal echocardiography due to cardio-oncologic surveillance during Herceptin treatment. She had a previous medical history of asthma and hypercholesterolaemia. In the 70 s, she had been diagnosed with Barlow’s mitral valve disease and a slightly dilated basal part of the pulmonary artery. In the past years, she reported heart palpitations and slightly volatile blood pressure levels. In ambulatory Holter monitoring, she had one non-sustained monomorphic ventricular tachycardia run but otherwise had a normal frequency of ventricular ectopic beats.

In echocardiography, she had a left ventricular (LV) ejection fraction of 55%. There was minimal regurgitation in the mitral valve. The inferoseptal wall had a long protrusion to the right ventricle, which was 4 cm long and 1 cm in width (*Panels A* and *D* and Supplementary data online, *Video S1*). The LV pouching filled up with blood during diastole and contracted normally during systole, ejecting the blood [*Panels B* and *C* and Supplementary data online, *Video S2* (50% slowed down)]. A 3D echocardiogram image was also captured (*Panel E*). There was no shunt communication to the right ventricle. An oncologic whole-body computed tomography had previously been performed and demonstrated imaging correlation with the echocardiography (*Panel F*).

The differential diagnoses of LV protrusion include aneurysm, pseudoaneurysm, diverticulum, cleft, and hypertrabeculation. Normal symmetric contraction ruled out an aneurysm or pseudoaneurysm. There were no signs of abnormal trabeculation. Considering the myocardium's normal function in the protrusion, the diagnosis of LV diverticulum was made. Although, the term double-chambered LV has been suggested as a more appropriate term for a diverticulum of this size. The true incidence of this rare incidental finding is currently unknown.

**Figure:**
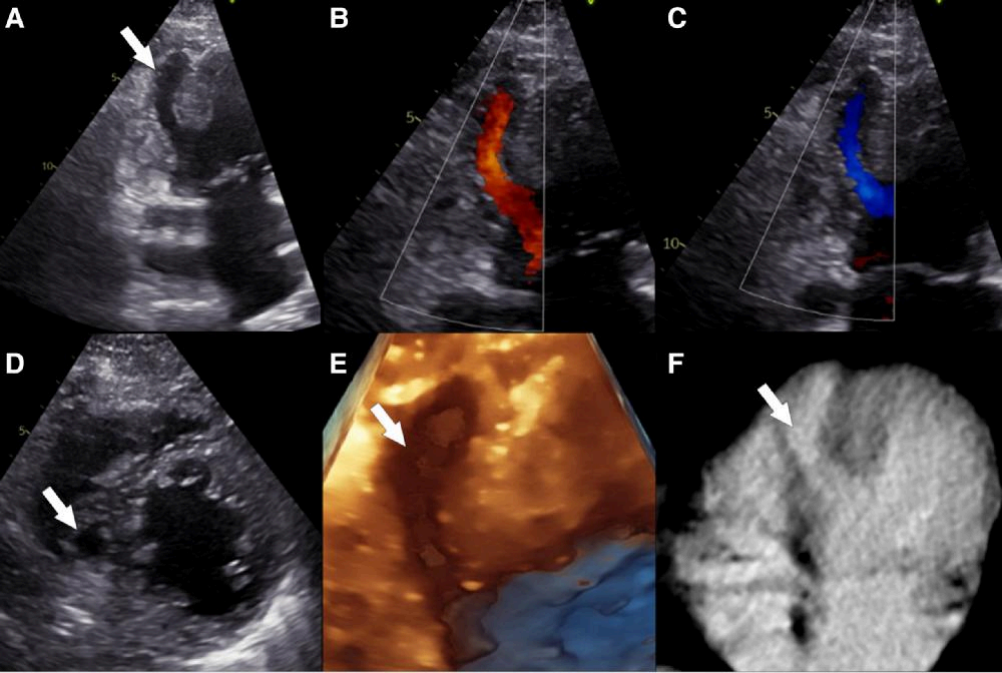


Supplementary data are available at *European Heart Journal - Cardiovascular Imaging online.*

**Funding:** None declared.

**Data availability:** The data underlying this article are available in the article and in its online supplementary material.

## Supplementary Material

jeaf103_Supplementary_Data

