# Supplementary figures and images for: Double-chambered left ventricle: a rare incidental finding on echocardiography
Source: Eur Heart J Cardiovasc Imaging. 2025 Apr 2;26(7):1313. doi: 10.1093/ehjci/jeaf103 (PMC12206578; doi:10.1093/ehjci/jeaf103)

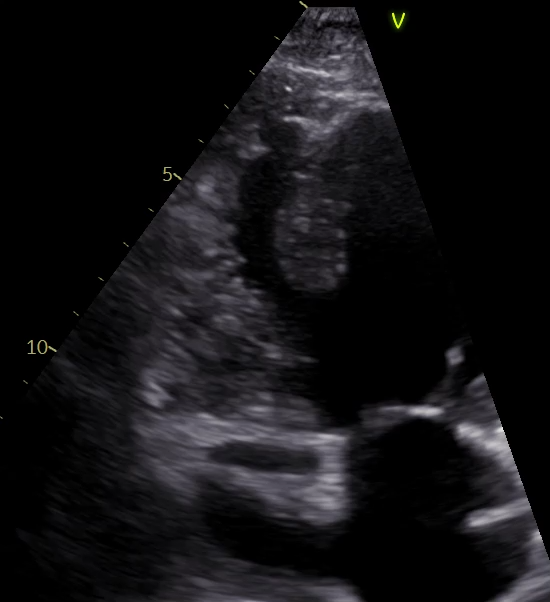

Supplement: jeaf103_Supplementary_Data [file jeaf103_supplementary_data.zip › Video 1 still image.png]

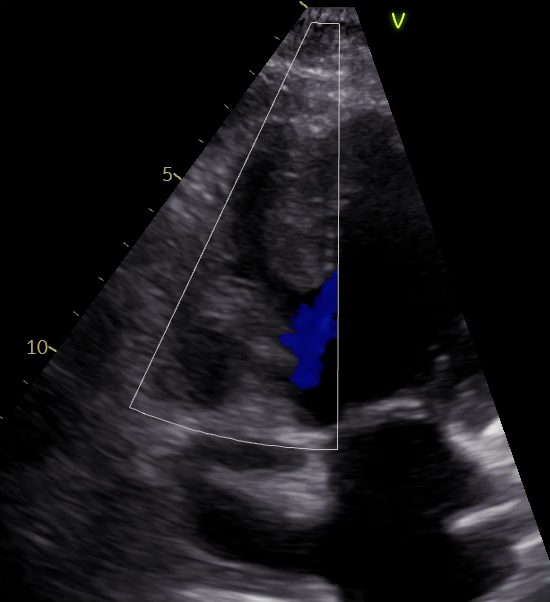

Supplement: jeaf103_Supplementary_Data [file jeaf103_supplementary_data.zip › Video 2 still image.png]
